# Toolkit of methodological resources to conduct systematic reviews

**DOI:** 10.12688/f1000research.22032.3

**Published:** 2020-10-14

**Authors:** Marta Roqué, Laura Martínez García, Ivan Solà, Pablo Alonso-Coello, Xavier Bonfill, Javier Zamora

**Affiliations:** 1 Iberoamerican Cochrane Centre - Sant Pau Biomedical Research Institute (IIB-Sant Pau), Barcelona, Spain; 2 CIBER of Epidemiology and Public Health (CIBERESP), Madrid, Spain; 3 Autonomous University of Barcelona, Bellaterra, Spain; 4 Clinical Biostatistics Unit, Ramón y Cajal Health Research Institute, Madrid, Spain

**Keywords:** Systematic reviews, prevalence, prognostic, diagnostic accuracy, efficacy of interventions

## Abstract

**Background: **Systematic reviews (SR) can be classified by type depending on the research question they are based on. This work identifies and describes the most relevant methodological resources to conduct high-quality reviews that answer health care questions regarding prevalence, prognosis, diagnostic accuracy and effects of interventions.

**Methods: **Methodological resources have been identified from literature searches and consulting guidelines from institutions that develop SRs. The selected resources are organized by type of SR, and stage of development of the review (formulation of the research question, development of the protocol, literature search, risk of bias assessment, synthesis of findings, assessment of the quality of evidence, and report of SR results and conclusions).

**Results: **Although the different types of SRs are developed following the same steps, each SR type requires specific methods, differing in characteristics and complexity. The extent of methodological development varies by type of SR, with more solid guidelines available for diagnostic accuracy and effects of interventions SRs.

This methodological toolkit describes the most up-to-date risk of bias instruments: Quality in Prognostic Studies (QUIPS) tool and Prediction model study Risk Of Bias Assessment Tool (PROBAST) for prognostic SRs, Quality assessment of diagnostic accuracy studies tool (QUADAS-2) for diagnostic accuracy SRs, Cochrane risk of bias tool (ROB-2) and Risk of bias in non-randomised studies of interventions studies tool (ROBINS-I) for effects of interventions SRs, as well as the latest developments on the Grading of Recommendations Assessment, Development and Evaluation (GRADE) system.

**Conclusions**: This structured compilation of the best methodological resources for each type of SR may prove to be a very useful tool for those researchers that wish to develop SRs or conduct methodological research works on SRs

## Introduction

Systematic reviews (SR) are studies that use a systematic and explicit method to identify, analyse and synthesize empirical evidence, and to answer a specific research question ^1^. Therefore, SRs are key tools to make informed health choices ^2, 3^.

All SRs are based on a specific research question. Classic epidemiological research questions relate to the prevalence of a medical condition, the associated prognosis of the medical condition (including incidence or global prognosis, prognostic factors associated to the condition's incidence or outcome, and risk profiles defined by prognostic models ^4^), diagnostic accuracy of tests that allow us to diagnose the medical condition, and effects of interventions to treat the medical condition. SRs can be classified by the type of research question they answer, as shown in Table 1.

**Table 1.** Research question by type of systematic review.

Type of systematic review	Acronym for the research question	Example of research question
**Prevalence review**	**CoCoPop-S** ( *condition, context, population and* *study design*)	What is the prevalence of frailty and prefrailty (condition) in community- dwelling older adults (population) living in low- and middle-income countries (context)? ^5^ What is the worldwide (population) prevalence of insufficient physical activity (condition) ^6^
**Prognostic review -** **global prognosis**	**CoCoPop-S** ( *condition, context, population and* *study design*)	What is the incidence of dementia (condition) in individuals of at least 60 years of age (population) living in high-income countries (context)? ^7^
**Prognostic review-** **prognostic factors**	**PICOT-S** ( *population, intervention or factor,* *comparison, outcome, time and* *study design*) **PFO-S** ( *population, factor or model,* *outcome and study design*)	Is protease activity (prognostic factor) an independent prognostic factor for wound healing (outcome) at 24 weeks (timeframe) in people with venous leg ulcers (population)? ^8^
**Prognostic review-** **prognostic models**	**PICOT-S** ( *population, intervention or factor,* *comparison, outcome, time and* *study design*)	What is best prognostic model to predict overall or progression-free survival (outcome) in patients with chronic lymphocytic leukaemia (condition)? ^9^
**Diagnostic** **accuracy review**	**PIRD-S** ( *population, index test, reference* *test, diagnosis of interest and study* *design*)	Do self-reported frailty to predict survival in adults with bacterial meningitis screening instruments (index test) accurately identify older people (population) at risk of frailty and prefrailty (condition of interest)? ^10^ Is PET 18F florbetapen (index test) useful in early diagnosing dementia (condition) in patients with mild cognitive impairment (population)? ^11^
**Effects of** **intervention** **review**	**PICO-S** ( *population, intervention,* *comparison, outcome of interest* *and study design*)	What is the effect of ribavirin (intervention) in patients with Crimean Congo haemorrhagic fever to prevent death (outcome)? ^12^ Does comprehensive geriatric assessment (intervention) in older adults (population) reduce mortality (outcome)? ^13^

The stages to develop an SR are common to all the types of SRs: 1) Formulating the research question, 2) development of the protocol that explicitly describes the methods to carry out each step of the SR, 3) literature search, 4) risk of bias assessment, 5) synthesis of findings, 6) assessment of the quality of evidence, and 7) report of SR results and conclusions ^1^. Although the different types of SRs share the same structure and follow a similar development process, their methods can be different and more or less complex depending on the type of SR.

Nowadays there are numerous methodological resources to conduct reviews, especially for intervention SRs and diagnostic SRs. However, the scattering of these resources and the lack of widely established manuals or recommendations are, in many situations, an obstacle to access them, especially for prevalence SRs and prognostic SRs. Therefore, the objective of this review is to identify and describe the methodological resources available to develop prevalence SRs, prognostic SRs, diagnostic accuracy SRs and effects of interventions SRs.

## Methods

### Information sources and search strategy

We consulted the guidelines from the main organizations that establish methods to conduct SRs ( Cochrane, Joanna Briggs Institute, European Network for Health Technology Assessment (EUNETHTA), Enhancing the Quality and Transparency of Health Research (EQUATOR) network, Grading of Recommendations Assessment, Development and Evaluation (GRADE)) in order to identify their proposed resources.

Additionally, we performed a literature search in MEDLINE (accessed through PubMed) in November 2019 using the following search syntax: ((“Review Literature as Topic”[Mesh] OR systematic review*[tiab] ) AND (handbook*[ti] OR methodolog*[ti] OR manual[ti] OR guide[ti]).

We also performed ad hoc scientific literature searches to find other resources for each type of SR in relation to the research question structure, the literature search strategy, the risk of bias assessment and the statistical analysis.

### Eligibility criteria

We included the resources available to design prevalence SRs, prognostic SRs, diagnostic SRs and intervention SRs.

We excluded the methodological resources to develop other types of SRs (methodological, economic evaluation and qualitative research SRs, or overviews).

### Data selection and extraction

The authors are members of CIBERESP (Centro de Investigación Biomédica en Red de Epidemiología y Salud Pública - Biomedical Research Center Network of Epidemiology and Public Health), hold active roles within Cochrane and the GRADE Working Group, and are experts in different fields of knowledge (statistics, development of Cochrane reviews, research methodology, information retrieval, development of clinical guidelines). They evaluated the search results, selected the most relevant and accurate resources, and summarized the most relevant information by development stage and type of SR.

The resources were selected based on the authors expert judgement, prioritising those resources which were endorsed or part of a guideline from the organisations cited above, and those which were more recent. The resources were organised in 7 sections, following the development stages of an SR: 1) Formulating the research question, 2) development of the protocol and review registration, 3) search strategy, 4) risk of bias assessment, 5) statistical synthesis of findings, 6) quality of evidence assessment, and 7) results report and presentation. The resources are presented by type of SR in each section, and an example of their use is included ^5– 13^.

For each pre-defined section, the authors selected and summarized the methods that were considered to be more rigorous and widely accepted, prioritizing major methods applicable to all reviews over more controversial methods, or methods which required highly specialized knowledge. The text organises the results pedagogically with the aim to highlight key differences between review types, present the key characteristics of each method, and be a comprehensive tool that contains the most relevant advice based on the authors judgement.

## Results

We identified guidance handbooks, primary studies and reporting guidelines as a result of the bibliographic searches. The resources selected are presented in Table 2.

**Table 2.** Organisation of resources by type.

Best practice manuals and chapters of manuals	Primary methods	Reporting guidelines
JBI manual Aromataris 2017 ^14^ (Munn 2017 ^15^ Moola 2017 ^16^ Campbell 2017 ^31^) Cochrane DTA manual Deeks 2010 ^20^ (Bossuyt 2008 ^24^ deVet 2008 ^32^ Macaskill 2010 ^60^) Cochrane intervention manual Higgins 2019 ^1^ (Lefebvre 2019 ^29^ Higgins 2019 ^55^ Deeks 2019 ^58^ Chaimani 2019 ^63^ Schünemann 2019 ^68^) GRADE Working group manual Schünemann 2013 ^64^ (Schünemann 2020 ^65^ Schünemann 2020 ^66^ Santesso 2019 ^67^) PROGRESS project Riley 2019 ^17^ Debray 2017 ^19^ Dekkers 2019 ^18^ manual	Hemingway 2013 ^4^ Munn 2018 ^21^ Iorio 2015 ^22^ Bossuyt 2006 ^23^ Lijmer 1999 ^25^ Strauss 2010 ^26^ Ge 2018 ^27^ Page 2018 ^28^ Atkinson 2015 ^30^ Lefebvre 2013 ^33^ Glanville 2006 ^34^ Wilczynski 2004 ^35^ Beynon. 2013 ^36^ Sampson 2011 ^37^ Bramer 2017 ^38^ Hartling 2016 ^39^ Glanville 2014 ^40^ Isojarvi 2018 ^41^ Horsley 2011 ^42^ Gentles 2016 ^43^ Hartling 2017 ^44^ Booth 2016 ^45^ Rethlefsen 2014 ^46^ Rethlefsen 2015 ^47^ Spencer 2018 ^48^ Hoy 2012 ^49^ Hayden 2013 ^50^ Morgan 2018 ^51^ Morgan 2019 ^52^ Wolff 2019 ^53^ Whiting 2011 ^54^ Sterne 2016 ^56^ Lau 1997 ^57^ Popay 2006 ^59^ Rutter 2001 ^61^ Rücker 2008 ^62^ Murad 2017 ^69^ Harder 2017 ^70^ Huguet 2013 ^71^ Campbell 2020 ^76^	Moher 2009 ^72^ Moher 2015 ^73^ Beller 2013 ^74^ Zorzela 2016 ^75^ McInnes 2018 ^77^ Moher 2007 ^78^ Page 2016 ^79^ Salameh 2019 ^80^

We have identified methodological guidelines dedicated to the development of prevalence SRs ^14^, global prognosis ^15^, and prognostic factor SRs ^16– 18^.

During the performed search, we identified methodological manuals to develop prognostic model SRs in the series of publications from the PROGRESS project ^19^, and in the resource compilation from Cochrane's Prognosis Methods Group.

For diagnostic accuracy SRs and effects of interventions SRs, we have identified the methodological manuals developed by Cochrane Collaboration are available ^1, 20^. The recommendations drawn from the guidance handbooks identified are complemented whenever necessary with specific primary method studies identified in our search.

### Formulating the research question

The type of SR is determined by the **research question**, which must be formulated in a structured manner as shown in Table 1. Careful development of the research question is vital, since the SR inclusion criteria will stem from it.

***Prevalence review.*** Prevalence SRs aim to answer the question “How common is a health problem in a specific population?” Prevalence SRs focus on existing cases at a given time, measure the global burden of a health problem, and describe the characteristics of the affected population, the geographical distribution of that problem and its variation among subgroups. The structure of the research question must include the elements of condition, context, population and study design (CoCoPop-S) ^21^, as shown in Table 1. The most adequate study designs to estimate the prevalence would be population registers or cross-sectional studies that include population-representative samples. For instance, Guthold *et al.* (2018) considers studies based on population surveys as a reliable source of information to obtain global prevalence estimators of insufficient physical activity ^6^.

***Prognostic review.*** SRs of prognosis are mainly based on three types of research questions: 1) “What is the risk of an specific population to have a health problem?”, descriptive question (review of global prognosis) that focuses in new cases occurring within a period of time (incidence), 2) “what factors are associated with or determine a specific outcome?”, an explanatory question (review of prognostic factors), and 3) “are there risk profiles that have higher probability of presenting specific outcomes?”, a result prediction question (review of prognostic models or risk prediction). We have excluded from the aim of this project a 4 ^th^ type of prognostic question, known as stratified medicine, and that alludes to the use of prognostic information to individualise therapeutic choices in a group of people with similar characteristics ^4^.

Structured questions about global prognosis must specify population, outcome, condition to be predicted, context and time frame to determine the incidence (CoCoPop-S). The study designs that provide more reliable incidence estimates are prospective cohort studies with representative samples ^15, 22^. Structured questions regarding either prognostic factors or models must include population; exposure in terms of the prognostic factor or model of interest, including how it is measured, the intensity and the exposure time; outcome, condition to be predicted; follow-up time; and context (PICOT-S or PFO-S) ^19, 21^. The best study designs to evaluate prognostic factors or models are also prospective cohort studies. For instance, Westby *et al.* (2018) published a prognostic factor SR that gives priority to the inclusion of cohort studies and, if none is found, it resorts to including case-control studies, which also explore the association of prognostic factors with the outcome of interest, although with less reliability ^8^.

***Diagnostic accuracy review.*** Diagnostic SRs aim to answer the question “How good is a test to identify or dismiss the presence of a condition or health problem in a particular population, in comparison with a reference test?” The research question can be posed with the elements of population, index test, reference test, diagnosis of interest and study design (PIRD-S) ^21^. The SR approach will depend on the role of the index test in the clinical diagnostic pathway: if it replaces another test, if it will be used in addition to another test to refine the diagnosis, or if it is a triage test previous to other tests ^23, 24^.

Diagnostic SRs preferentially include cross-sectional studies, where the participants are evaluated using the index test and/or the reference test to determine if they have the condition of interest. Case-control designs are subject to risk of bias and their inclusion in diagnostic SRs is not recommended ^25^. For instance, Ambagtsheer *et al.* (2017) include in their SR cross-sectional studies where one or more self-reported frailty screening scales have been compared with one of three reference standards: frailty phenotype, frailty index or comprehensive geriatric assessment ^10^.

***Effects of interventions review*** Interventions SRs aim to answer the question “What effect does a specific intervention have on the relevant outcomes in people with a particular health problem, in comparison with a reference intervention?” The research question is posed with the elements of population, intervention, comparator, outcomes of interest and study design (PICO-S) ^1^.

The randomised clinical trial (RCT) is the most appropriate study design to evaluate the effects of an intervention, as it is the design with less risk of bias and that best helps to establish causality. In cases where it is not possible to conduct randomised trials for ethical or organizational reasons, non-randomised trials, before-after studies, time series, cohort studies or case-control studies can be considered for their inclusion in the SR ^1^. For instance, the SR by Johnson *et al*. (2018) regarding ribavirin for treating Crimean Congo haemorrhagic fever included both RCTs and non-randomised trials to use the available data, given the previous lack of preparedness for experimental research therapeutics in outbreak situations, but concludes that estimates of effect based on the existing literature are highly uncertain due to confounding in non-randomised studies ^12^.

### Development of the protocol and review registration

Writing the SR protocol is a fundamental step that must be done before designing an SR. Herein, the stages and methods to be applied during the development of the SR can be pre-specified. The identified guidelines can be used to identify the methods that need to be stated in the protocol, and some have specific chapters on protocol development ^1, 14, 20^. 

Similarly to the requirement of clinical trial registration, the SR should also be registered in order to avoid redundancies and, more importantly, to avoid reporting bias, therefore guaranteeing transparency and rigor during the development of the SR ^26^. Prospective registration of an SR protocol is recommended by the PRISMA guidelines and is associated with higher SR methodological quality ^27^. The largest and most well-known SR register is PROSPERO, produced by the Centre for Reviews and Dissemination in York. With PROSPERO, it is possible to prospectively register any type of review, provided that its aim is a health-related outcome. It contains more than 30,000 entries ^28^. All Cochrane SR protocols are published in Cochrane Library and automatically registered in PROSPERO.

### Search strategy

Designing a comprehensive research study for an SR is vital in order to reduce bias when identifying studies, and it is important to describe it in the relevant section within the protocol in a transparent and thorough manner to facilitate its evaluation by third parties and its reproducibility.

Methodological reference standards to design comprehensive searches have been published ^29, 30^. In addition, methodological manuals to develop SRs provide guidelines for diagnostic and effects of interventions SRs ^31– 33^.

The design of the search strategies does not differ by type of SR, but rather their differences are due to the elements of the research question and the design of studies to be identified. In general terms, electronic searches are designed to identify bibliographic references that use a language similar to the elements of the review's clinical question. To this effect, the strategies are built based on the elements of the structured clinical question. Search algorithms use a combination of natural language and the appropriate controlled vocabulary for each bibliographic database. Validated filters can be applied to these strategies to determine specific study designs that can be useful to identify, among others, clinical trials ^32– 34^, or prognostic studies ^35^. However, the use of filters is controversial in diagnostic accuracy studies ^32, 36^.

Search performance will vary depending on the type of studies that are included in the SR. Thus, in intervention SRs, the search results for RCTs are more precise (they have a higher proportion of relevant references among all the references that the search has identified), due to better indexation of this type of studies in bibliographic databases. On the contrary, in SRs that include observational studies, like prognostic SRs, identifying studies is more complex given the variability of designs to be included and its poorer indexation in databases, which results in less specific literature searches that lead to a longer and more complex study selection process ^17^.

Searches must be designed to optimise their sensitivity (the ability to retrieve as many relevant study references as possible), which is a feature that tends to be a detriment to precision, which in SRs ranges on an average of 3% ^37^. To obtain an efficient search with adequate sensitivity, performing searches in MEDLINE and EMBASE may be sufficient, particularly in intervention reviews, as they are the two most frequently used bibliographic databases ^38^, and they are enough to identify most relevant studies for a specific SR ^39^. These searches can be complemented with additional searches in other databases such as PEDro, which provide specific information for certain topics.

Searching in bibliographic databases can be completed with additional strategies, such as checking public trial registers ^40, 41^, searching in the reference list of relevant studies ^42^, or cross-searching citations ^43^. Searching grey literature, understood as any document that is not published in biomedical or scientific journals, has a limited impact in effects of interventions SRs ^44^, but offers good results in other types of SRs, such as qualitative evaluation SRs ^45^.

If we take into consideration the methodological and technical challenges that the design and implementation of search strategies pose, involving a medical librarian can be desirable to improve the search quality ^46– 48^.

### Risk of bias assessment

Assessing the risk of bias of the included studies is a key element in any SR. It helps evaluate and interpret the included studies results, and it is a determinant of the evidence quality of the SR results. The current tools to assess risk of bias are organised by domains, which roughly correspond to the classic epidemiological biases related to each type of research question. The identified tools to assess risk of bias are presented in Table 3, organised by type of SR and by domain of epidemiological bias assessed.

**Table 3.** Tools to assess risk of bias by type of systematic review.

	Scale (n items)	Selection bias (number of items)	Exposure and performance bias (number of items)	Outcome detection bias (number of items)	Attrition bias(number of items)	Confounder bias (number of items)	Selective outcome reporting bias (number of items)	Other biases (number of items)
**Prevalence** **review**	**Hoy 2012** **(10) ^49^**	- Representativeness of population sample (1) - Sample and recruitment (2)	(0)	- Data collection (2) - Case definition and timeframe for prevalence (2) - Reliability of measuring instrument (1)	- Impact of missing data (1)	(0)	(0)	- Appropriate computation of prevalence estimator (1)
**Prognostic** **review-** **prognostic** **factors**	**QUIPS (31) ^50^**	- Study participation (3) - Sample and recruitment (3)	- Prognostic factors definition and measurement (6) - Confounders definition and measurement (4)	- Outcome definition and measurement (3)	- Description and impact of attrition (6)	- Statistical analysis of confounding factors (2)	- Selective reporting of results (1)	- Statistical analysis (3)
	**RoB for** **NRS -** **exposures** **(32) ^52^**	- Selection of participants (5)	- Exposure definition and measurement (5) - Deviations from intended exposure (4)	- Outcome definition and measurement (5)	- Description and impact of attrition (5)	- Statistical analysis of confounding factors (6)	- Selective reporting of results (3)	(0)
**Prognostic** **review-** **prognostic** **models**	**PROBAST** **(20) ^53^**	- Design of study and selection of participants (2)	- Prognostic factors definition and measurement (3)	- Outcome definition and measurement (6)	- Inclusion of participants in the analysis (2)	(0)	- Selective reporting of results (1)	- Statistical analysis (6)
**Diagnostic** **accuracy** **review**	**QUADAS-2** **(11) ^54^**	- Selection of participants (3)	- Index test interpretation (1) - Threshold specification for index test (1)	- Adequacy and interpretation of reference test (2) - Time interval between tests, and coverage of reference test (3)	- Inclusion of participants in the analysis (1)	(0)	(0)	(0)
**Effects of** **intervention** **review**	**ROB-2 (16) ^55^**	- Selection of participants (randomisation, concealment, and basal imbalances) (3)	- Blinding of participants and personnel (2) - Deviations from intended intervention (2)	- Blinding of outcome detection (2)	- Impact of attrition (3)	(0)	- Selective reporting of results (2)	- Analysis of participants in the allocated intervention arm (2)
	**ROBINS-I** **(35) ^56^**	- Selection of participants (6)	- Classification of intervention (3) - Deviations from intended intervention (6)	- Outcome measurement (4)	- Description and impact of attrition (5)	- Confounders (8)	- Selective reporting of results (3)	(0)

No risk of bias tool has been identified for global prognosis systematic reviews. The number of items in the risk of bias tools may vary depending on the effect of interest and the included study designs, as well as the addition or suppression of index questions by the researchers to tailor the tool to the SR.

Each of the domains of these tools includes a number of index questions related to specific aspects of study design or development that can lead to a bias in that domain. The tools can be adapted *a priori* to each review, modifying or deleting questions, or adding new questions specific to the considered research question. The process to assess risk of bias is similar in all the current scales. Firstly, they identify the risk of bias in each domain based on the answers to the questions, and secondly, they integrate these risks in a risk of bias assessment for each health problem, prognostic factor, diagnosed condition or outcome of interest assessed, depending on the type of SR.

***Prevalence review.*** The tool to assess risk of bias by Hoy *et al.* (2012) is available for prevalence SRs. It assesses internal and external validity aspects in the prevalence study ^49^. The tool comprises 10 questions where a judgement of high or low risk of bias is made. Based on the answers, the researcher makes a subjective assessment of the study’s overall risk of bias as low, moderate or high ^49^.

***Prognostic review.*** There is no scale available to assess the risk of bias in **global prognostic studies**, although a series of criteria has been proposed to assess risk of bias. These are classified in 1) definition and representativeness of the population, 2) completeness of follow-up, and 3) objective and unbiased measurement of outcome of interest ^22^. However, some authors like Roerh *et al.* (2018) use a version of the scale to assess risk of bias designed by Hoy *et al.* (2012), adapted to the assessment of incidence studies considering the duration of the incidence period ^7^.

For the **prognostic factor** studies, the tools QUIPS and “RoB instrument for NRS of exposures” were identified ^50– 52^. The QUIPS tool helps assess the risk of bias using 31 questions divided in 6 domains. For each domain, a judgement of high, low or unclear risk of bias is made. Before using the tool, one must carefully consider the potential confounders that can lead to bias. Clinical experts in the specific topic of the SR should participate. The tool “RoB instrument for NRS of exposures” evaluates the risk of bias using 32 questions divided in 7 domains, including a key domain regarding confounders and a domain regarding departures from intended exposures. For each domain, a judgement of critical, serious, moderate or low risk of bias is made. An example of the use of the QUIPS scale can be seen in the review by Westby *et al*. (2018). The authors defined *a priori* two key confounders (age and infection), which the experts and the literature described as prognostic factors for their condition of interest (venous leg ulcers), and which were simultaneously associated with the prognostic factor of interest in the SR (protease activity biomarker). These two confounders were included in the QUIPS scale in the section of control by confounders ^8^.

We identified the Prediction model Risk Of Bias ASsessment Tool (PROBAST) for the **prognostic** model SRs ^53^. This tool assesses the risk of bias using 20 questions divided in 4 domains (participants, predictors, outcome and analysis). For each domain, a judgement of high, low or unclear risk of bias is made. The questions vary according to the aim of the study (development, validation, or development and validation of the prognostic model).

***Diagnostic accuracy review*** The tool QUADAS-2, which evaluates 11 questions divided in 4 domains, is available to assess the risk of bias in diagnostic accuracy studies ^54^. For each domain, a judgement of high, low or unclear risk of bias is made. In addition, the external validity or study applicability in relation to the SR is assessed in each domain.

Diagnostic SRs mainly include observational studies, which are more subject to risk of bias, and therefore adapting the QUADAS-2 tool, modifying or adding specific questions to the SR topic, is virtually a requirement during the protocol stage. For instance, the SR by Martínez *et al*. (2017) studied the diagnostic accuracy of an imaging test (amyloid PET) that requires complex visual interpretation. For this reason, a question was included in the QUADAS scale to assess whether the test interpretation was performed by trained readers ^11^.

***Effects of interventions review.*** For intervention SRs, the Risk of Bias (RoB) 2.0 tool is available to assess the potential bias in randomised clinical trials, and the Risk Of Bias In Non-randomised Studies - of Interventions (RoBiNS-I) tool in non-randomised clinical trials ^55, 56^. The RoB 2.0 tool includes 16 questions divided in 5 domains, including a specific domain for randomisation and a domain for deviations from intended interventions ^55^. The number of questions may vary, depending on the effect of interest and the design of the study assessed. For each domain, a judgement is made: high or low risk of bias, or some concerns. For instance, in their SR, Ellis *et al.* (2017) assessed the risk of bias in the evaluation of results separately for the objective outcomes (such as living at home or death) and for the subjective outcomes, showing a lower risk of bias in the evaluation of the objective outcomes ^13^.

The RoBiNS-I tool assesses the biases that the non-randomised study has when compared with an ideal, pragmatic, unbiased randomised trial, which answers the clinical question of interest (even if this ideal trial may not be feasible or ethical) ^56^. RoBiNS-I has 34 questions divided in 7 domains, including a key domain regarding confounders and a domain for deviations from intended interventions. As in the case of prognostic SRs, there should be an *a priori* careful consideration of the potential confounders that must be included in the tool to assess individual studies. A judgement of critical, serious, moderate or low risk of bias is made for each domain. A low risk of bias implies that the non-randomised study is comparable to a well-performed randomised trial. For instance, Johnson *et al.* (2018) excluded from their analyses the non-randomised studies that showed a critical risk of bias according to RoBiNS-I, rejecting 18 out of the 22 included studies ^12^.

### Statistical synthesis of findings

SRs may include a section with a quantitative statistical synthesis or meta-analysis, where a combined estimator of the parameter of interest is obtained from the estimators of the individual studies. Table 4 shows a non-exhaustive compilation of the main characteristics of the meta-analysis methods and the main software commands for each type of SR.

**Table 4.** Methodological characteristics of meta-analysis by type of systematic review.

	Measures to combine	Assessment of heterogeneity	Model	Method	Command (package)
Prevalence review	- Proportion (prevalence)	- Qualitative	- Fixed/Random effects	- Inverse-variance method a	- Metaprop (Stata)
Prognostic review - global prognosis	- Cumulative incidence - Incidence rate	- Meta-regression	- Fixed/Random effects	- Inverse-variance method b	- Metan (Stata) - Metaprop (Stata) - Review Manager
Prognostic review- prognostic factors	- Hazard Ratio - Odds Ratio	- Meta-regression	- Random effects	- Inverse-variance method	- Metafor (R)
Prognostic review- prognostic models	- Calibration - Discrimination	- Meta-regression	- Random effects	- Multivariate methods	- Metamisc (R)
Diagnostic accuracy review	- Sensitivity - Specificity	- Meta-regression	- Random effects	- HSROC method c - Bivariate model	- Metadas (SAS) - Metandi (Stata)
Effects of intervention review	- Mean difference - Risk difference - Standardised mean difference - Hazard Ratio - Incidence rate ratio - Odds Ratio - Risk ratio	- I ^2^ - Meta-regression	- Fixed/Random effects	- Mantel-Haenzsel method - Multivariate methods	- Metafor (R) - Metan (Stata) - Review Manager

^a^ Tukey-Freeman or logit transformation. ^b^Transformation for the cumulative incidence. ^c^ Hierarchical summary receiver operating characteristic (HSROC) method allows estimation of a Receiver operating characteristic (ROC) curve or sensitivity and specificity indexes.

A necessary previous step to any meta-analysis is the evaluation of the existing clinical and statistical heterogeneity in the set of studies, which will inform us 1) if it is reasonable to perform a quantitative synthesis of findings, 2) what meta-analysis model we should apply, and 3) if additional investigation of the causes of heterogeneity is required, for example, subgroup and sensitivity analyses, or meta-regressions ^57, 58^. In those cases when a quantitative synthesis is precluded, the SR will be restricted to a narrative synthesis. A narrative synthesis should not simply summarize the findings from the included studies in order to draw conclusions about the body of evidence, but instead should be a more formal process which includes a formulation of the theory of how the intervention works, why and for whom, the exploration of the relationships in the data, and the assessment of the robustness of the synthesis ^59^.

When it is reasonable to perform a statistical synthesis, there are two main models to conduct a meta-analysis: fixed effects model and random effects model. For practical purposes, the chosen model determines how the studies included in the meta-analysis will be numerically weighed. Both models are based on different assumptions regarding distribution of effects and heterogeneity in the set of studies, and they differ in their application and interpretation ^58^.

Finally, there is a variety of resources to conduct meta-analyses, from specific programs to perform meta-analyses (free or paid) to user-defined routines using general statistics packages (SAS, Stata, SPSS), as well as Excel utilities or R libraries. An archive with software and utilities is available from SR Tool Box.

Due to the complexity of the statistical techniques to synthesise results, and the difficulty to standardise methods and decisions to be made during the analysis, it is vital to involve a statistician in the planning and conduct stages of the meta-analysis, especially for prognostic and diagnostic SRs.

***Prevalence review.*** In prevalence SRs, the meta-analysis combines ratios, which are transformed to be meta-analysed using the inverse-variance method ^58^. Siriwardhana *et al.* (2018) calculated combined frailty prevalence estimates using a random effects model. The authors assessed that there was high clinical heterogeneity between the studies in terms of actual frailty prevalence, geographic setting, frailty assessment method, cut-off points applied and sample age, although this heterogeneity did not rule out performing a meta-analysis ^5^.

***Prognostic review.*** In global prognostic SRs, the meta-analysis combines cumulative incidence ratios or incidence rates, while in prognostic factor SRs, the meta-analysis combines odds ratios or hazard ratios, which can be presented in individual studies as raw estimates or as covariate-adjusted estimations derived from logistic or Cox regression models. If combining adjusted estimates, all of them should be adjusted by a minimum set of common factors ^17^. In prognostic model SRs, the meta-analysis combines estimates of model discrimination and calibration. These indicators can be synthesised separately or jointly using multivariate models ^19^.

Prognostic studies usually show significant variability in terms of design, sample case-mix, measurement instruments, analysis methods and presentation of results ^17^. Therefore, in prognostic factor and model SRs, it is recommended to perform the meta-analysis using the random effects model, and even to use multivariate meta-analysis methods adjusting for relevant factors ^17^. For instance, the SR by Westby *et al.* (2018) describes how the authors dismissed performing a meta-analysis due to the high risk of bias and the extreme heterogeneity across the included studies in terms of population, measurement of the prognostic factor (cut-off points and analytical methods) and outcome measurement ^8^.

***Diagnostic accuracy review.*** In diagnostic SRs, the meta-analysis combines estimates of sensitivity and specificity of the index test. The meta-analysis in diagnostic SRs shows a higher degree of complexity because the studies may have used different thresholds, both implicit and explicit, to define a positive result in the evaluated test. This leads to a correlation between the sensitivity and specificity indexes, which must be modelled jointly using multivariate methods ^60^. The most common available statistical methods are the bivariate hierarchical model and the HSROC model (Hierarchical summary receiver-operating characteristic) ^61^. Diagnostic SRs tend to combine studies with very heterogeneous results, and it is recommended to use the random effects model by default and perform a comprehensive examination of the sources of heterogeneity using meta-regression ^60^. For instance, the protocol of the SR by Ambagtsheer *et al.* (2017) expects to estimate an average sensitivity and specificity for the frailty scales, when the included studies have applied the same explicit cut-off points to the considered scales. However, given that they are subjective, self-reported scales, the studies could share the same explicit cut-off point, and yet that cut-off point could correspond to different levels of frailty in the studies (implicit thresholds), which will advise against calculating pooled estimates of diagnostic accuracy ^10^.

***Effects of interventions review.*** In intervention SRs, the meta-analysis combines different measures, depending on the type of outcome: odds ratio or risk ratio for binary outcomes, mean difference or standardised mean difference for continuous outcomes, hazard ratio for time-to-event outcomes, and incidence rate ratios for outcomes that count number of events.

In intervention SRs, the I ^2^ estimator has been proposed to assess statistical heterogeneity as a supplement to the assessment of clinical and methodological heterogeneity. This indicator is defined as the percentage of the overall variability that cannot be explained by chance, and has values ranging from 0% to 100%; with higher values indicating higher statistical heterogeneity ^58^. For instance, I ^2^ was one of the aspects considered in the SR by Ellis *et al.* (2017) to assess the inconsistency in results, and to decide if a meta-analysis combining the results would be performed ^13^. Despite its popularity and ease of interpretation, the use of this indicator is not exempt of controversy due to its dependence on the number of studies and sample size; thus, a small statistical heterogeneity could seem substantial only by the effect of a large sample size of the included studies ^62^.

In intervention SRs, pairwise metanalysis has been extended to network metanalysis, which allows the simultaneous comparison of three or more interventions, combining direct and indirect evidence from a network of studies ^63^.

### Quality of evidence

The quality (also confidence or certainty) of evidence in an SR is the degree of confidence that is held against the fact that an estimate of effect or association is close to the actual value of interest ^1^. Certainty of evidence is best evaluated with the GRADE system. Certainty in the obtained estimates for each one of the key SR outcomes or factors is classified as high, moderate, low or very low. A level of certainty of evidence is first established from the design of the studies that form the evidence body, which might or might not have an optimal design for the type of considered question. This initial confidence in the evidence body can then decrease in one or two levels if the following is detected: 1) design or execution limitations, 2) inconsistency between estimates, 3) indirect evidence, 4) imprecision in estimates, or 5) publication bias ^64^.

The certainty of evidence is a key element to interpret and communicate results, and as such, it should be included in the sections of results, discussion, conclusion and abstract, using semi-standardised statements ^65^. Additionally, it can be included in a Summary of Findings table, where for each comparison, the key information regarding relative effect and absolute effect magnitude, quantity of available evidence and its certainty is presented ^66^. Certainty of evidence can be assessed too when no quantitative synthesis is possible ^67^.

We will now highlight the specific aspects in which the GRADE system adapts to each type of SR.

***Prevalence review.*** There are no formal adaptations of the GRADE system for prevalence SRs, but there is a proposal to assess the quality of the evidence based on this system ^68^. High initial certainty is awarded to survey or cross-sectional study designs with population representativeness that have been properly designed and conducted, while studies with no population representativeness will have lower initial quality.

***Prognostic review.*** There is a GRADE proposal for global prognostic SRs ^22^ and an adaptation for prognostic factor SR ^69^. Guidelines for prognostic model SR are still under development.

In **global prognostic** SRs, the study designs that have high initial certainty are longitudinal cohort studies and pragmatic randomised controlled trials with representative samples ^22^. Other observational designs would offer low initial certainty. In **prognostic factor** SRs, explanatory and confirmatory longitudinal designs offer high initial certainty, while exploratory studies are considered to be of moderate quality ^69^.

In prognostic SRs, the assessment of the limitations follows the general procedure already described, with two particularities: 1) qualitative assessment of inconsistency, because of low reliability of I ^2^ estimator in the prognostic field ^22, 69^, and 2) possibility of increased certainty in the studies that do not show limitations in the quality of evidence, if (i) the estimated effect magnitude is substantial, or (ii) there is an exposure-response gradient ^67^. For instance, the prognostic factor SR by Westby *et al.* (2018) considered the possibility of increasing the certainty of evidence in the studies presenting no limitations. Due to the exploratory nature of the included studies and their high risk of bias, the certainty was not increased in any case and the evidence obtained in the review was of very low quality ^8^.

***Diagnostic accuracy review.*** There is a GRADE proposal for assessing the certainty of evidence for test accuracy ^70, 71^. The study designs that start with the highest degree of evidence are cohort or cross-sectional studies where the index test and an appropriate reference standard have been directly compared in patients with diagnostic uncertainty ^70^. If the SR included case-control studies, these would offer low-quality initial evidence ^25^.

Indirectness of evidence would be assessed through any applicability concerns of the patient sample, the intervention and the comparator with respect to the clinical pathway where the test is to be applied. There is uncertainty regarding how to assess inconsistency, because heterogeneity is common and hard to quantify in diagnostic SRs, and it often cannot be explained even if multivariate models are adjusted. Judgments on extent of heterogeneity should be based on similarity of the point estimates, overlap of confidence intervals, and the exploration of possible explanations for the inconsistency from subgroup or sensitivity analyses ^70^.

Imprecision judgments should be based on both the width of the confidence or credible intervals for sensitivity and specificity, but also on the implications for patient management in terms of true and false positives, and true and false negatives. When the estimated intervals include values that may lead to different conclusions of the test’s value, the certainty of the evidence may be lowered ^71^.

With regard to the criteria to increase the level of evidence, it is unclear whether they should be applied at all and how to do it in diagnostic SRs ^71^. The uncertainty surrounding the process of assessing the quality of evidence in diagnostic SRs explains why it is not a requirement in Cochrane SRs at the moment. For instance, the SR by Martínez *et al.* (2017) only included a Summary of Findings table with numerical results and an estimation of the absolute effect that the test would have on a hypothetical cohort of individuals ^11^.

***Effects of interventions review.*** The GRADE system for assessing the quality of evidence was initially developed for intervention SRs, and it is the indication for which clearer and widely agreed guidelines are available ^64^. In terms of study design, RCTs are initially classified as having high certainty, while all non-randomised or observational studies are classified as having low certainty. This proposal for pairwise metanalysis can be extended to network metanalysis ^63^.

The assessment of the certainty limitations is well-defined in intervention SRs. Inconsistency can be assessed using the I ^2^ estimator ^64^. Imprecision is assessed taking into account whether the review meets the optimal information size, and whether the confidence interval of the effect estimate allows reaching a conclusion, because either it only includes values consistent with a relevant intervention effect, or it completely dismisses it ^63^. In observational studies that do not have limitations in the quality of evidence, three criteria are considered to increase certainty: 1) the estimated effect magnitude is important or very important, 2) there is an exposure-response gradient, and 3) all possible biases that could reduce the observed effect confirm the obtained conclusions.

For instance, the SR by Ellis *et al.* (2017) applied the GRADE system to the included randomised trials, and it concluded that there was high certainty of the effect of the comprehensive geriatric assessment on the effects outcomes based on a high number of studies and participants, with a globally low risk of bias, and results consistent among studies. However, the certainty of evidence obtained in cost-effectiveness was low, due to imprecision and inconsistency of results ^13^.

### Results report

It is vital to inform about the methods, results and conclusions of the SRs in a transparent and thorough manner so that their users can interpret, evaluate and apply them. The EQUATOR initiative has developed, and keeps up-to-date, a library with guidelines to communicate the different types of research studies. The PRISMA statement (Preferred Reporting Items for Systematic Reviews and Meta-Analyses) has been proposed in the SR field ^72^. This statement consists of a checklist comprised of 27 items and a flow diagram to present the number of studies considered in the SR. In addition, several extensions focusing on reporting specific aspects of SRs have been developed, such as PRISMA-P for reporting SR protocols ^73^, PRISMA- *Abstracts* for reporting abstracts ^74^, and PRISMA- *Harms* for reporting harms outcomes in SRs ^75^. Additionally, the SWiM guideline is available for reporting intervention SRs where the effects of interventions are synthethised narratively without metanalysis, focusing on the key features of narrative information synthesis (grouping of studies, presentation of data and summary text, and appropriate discussion of limitations of this type of synthesis) ^76^.

Although the PRISMA statement and the cited extensions are focused on intervention SRs, a specific PRISMA extension has also been developed for diagnostic SRs ^77^. On the contrary, no tools have been identified to communicate prevalence or prognostic SRs. In recent years, clarity and transparency in study communications has improved thanks to the development of checklists for scientific paper publication, although there is still room for improvement ^78– 80^.

## Discussion

### Key results

This review identifies and describes the most relevant methodological resources to conduct prevalence, prognostic, diagnostic accuracy and effects of interventions SRs. This review offers a general and comparative perspective of the methodological resources by SR stage, highlighting the differential elements of each type of SR. This project does not aim to be a standalone tool for a researcher to find complete guidance on how to conduct and report a review, but rather it aims to be a signpost pointing out to the resources where researchers may find in depth guidance to develop their reviews.

### Current context

This paper corroborates that developing a rigorous SR is a complex and resource-intensive task ^81, 82^. In order to tackle the increasing complexity of SRs and ensure the adoption of rigorous methodology, it is necessary that the reviews are made by **multidisciplinary work groups** with knowledge and experience in methodology (such as statistical analysis and information retrieval) ^83, 84^. In addition, it is important to consider the increasing availability of artificial-intelligence-based **technological tools**, which make it possible to semi-automate the different steps of the SR development, and thus reduce the time and human resources required to conduct the review ^85^.

Once the rigorous SR has been developed, ensuring the conveyance of the generated knowledge is essential. In this sense, new **formats for synthesis** and presentation of SR results are being explored nowadays to help their dissemination and the adoption of their conclusions in clinical practice and healthcare decision-making. For instance, new formats for result presentation and Summary of Findings tables are being proposed, adapted to the profile of their potential users ^86, 87^.

### Limitations and strengths

An inherent limitation of this project is its methodology based on a selection of resources and summary of guidance informed by expert opinion, which may be susceptible to implicit selection biases or lack of comprehensiveness.

The four types of SRs considered in this paper are fundamental to define preventive activities and public health policies, as well as to make health decisions. The selection of resources done is not dependent on whether the reviewer explores questions on efficacy or effectiveness, often described as explanatory or pragmatic questions, and will be useful to the researchers regardless of their intended purpose. However, we have not considered the resources to conduct in-depth exploration of effectiveness issues such as reviews of complex interventions or implementation reviews. Additionally, this research has not considered other types of SRs, such as methodological, economic evaluation and qualitative research SRs, for which it would be convenient to perform similar methodological compilations. Reviews of reviews (or overviews) were also not considered, and as such, there are a number of review-level resources which have not been discussed, for example the risk of bias assessment tool ROBIS or the methodological assessment tool AMSTAR ^88, 89^. Another limitation of this research is the need to keep it up to date, given the speed at which the methods and methodological resources to develop SRs are updated.

On the other hand, the main strengths of this paper are its transversal approach for the different types of reviews, and the identification of resources for all the stages in the development of an SR. There are few previous publications that offer a transversal perspective of the different types of systematic reviews, and these are focused on a specific stage of the review or on a particular topic. For instance, the work carried out by Munn *et al.* (2018) defined a typology for SRs, characterised from 10 different types of research questions, and delving into the format of each type of question ^21^. Pollock *et al.* (2017) review the steps of an SR for 5 types of question, specifically focusing on the particularities of the reviews on stroke rehabilitation ^90^. Muka *et al.* (2019) offer a structured compilation of resources for each SR stage, but without delving into the specificities of the different types of SRs ^91^. Finally, organising the resources to assess the risk of bias by type of review is a strength and a novelty compared with previous works, which compile the quality assessing tools by type of study design but without linking them to the aim of the study nor the type of systematic review ^92, 93^.

## Conclusions

SRs are a key research tool to make decisions in healthcare, public health and medical research. There are methods and resources to develop high-quality reviews to answer most types of clinical questions. This review offers a complete resource guide for prevalence, prognostic, diagnostic and intervention reviews, and is a very useful tool for those researchers that wish to develop SRs or conduct methodological research works in that field.

## Data availability

### Underlying data

All data underlying the results are available as part of the article and no additional source data are required.
