# Supplementary material for: Toolkit of methodological resources to conduct systematic reviews
Source: F1000Res. 2020 Oct 14;9:82. Originally published 2020 Feb 4. [Version 3] doi: 10.12688/f1000research.22032.3 (PMC7542253; doi:10.12688/f1000research.22032.3)
Supplement: Peer review history

# Peer review: Reviewer response for version 3

**DOI:** 10.5256/f1000research.30171.r73009

**Published:** 2020-10-15

**Authors:** Miranda Cumpston

**Affiliations:** 1 Monash University, Melbourne, Australia; 2 University of Newcastle, Newcastle, Australia

No further comments.

Is the work clearly and accurately presented and does it cite the current literature?

Yes

If applicable, is the statistical analysis and its interpretation appropriate?

Not applicable

Are all the source data underlying the results available to ensure full reproducibility?

No

Is the study design appropriate and is the work technically sound?

Yes

Are the conclusions drawn adequately supported by the results?

Yes

Are sufficient details of methods and analysis provided to allow replication by others?

No

Reviewer Expertise:

In presenting my comments, I acknowledge that my own experience focuses on methods around systematic reviews of the effects of interventions, and so I cannot comment with expertise on the details of methods described for other review types.

I confirm that I have read this submission and believe that I have an appropriate level of expertise to confirm that it is of an acceptable scientific standard.

# Peer review: Reviewer response for version 2

**DOI:** 10.5256/f1000research.28216.r69215

**Published:** 2020-10-07

**Authors:** Edward Purssell

**Affiliations:** 1 School of Health Sciences, City, University of London, London, UK

I would just push point 5 regarding heterogeneity. Cochrane is quite clear about this - stating "Thus, the test for heterogeneity is irrelevant to the choice of analysis; heterogeneity will always exist whether or not we happen to be able to detect it using a statistical test" ^1^. I think that this just needs a bit more explanation.

Is the work clearly and accurately presented and does it cite the current literature?

Yes

If applicable, is the statistical analysis and its interpretation appropriate?

Not applicable

Are all the source data underlying the results available to ensure full reproducibility?

No

Is the study design appropriate and is the work technically sound?

Yes

Are the conclusions drawn adequately supported by the results?

Yes

Are sufficient details of methods and analysis provided to allow replication by others?

Partly

Reviewer Expertise:

Paediatrics, infection control, systematic review.

I confirm that I have read this submission and believe that I have an appropriate level of expertise to confirm that it is of an acceptable scientific standard.

## References

[1] : Cochrane Handbook for Systematic Reviews of Interventions (Version 6.1).2020; Reference source

# Response

**Published:** 2020-10-09

**Authors:** Marta Roqué

**Affiliations:** 1- Iberoamerican Cochrane Centre - Sant Pau Biomedical Research Institute (IIB-Sant Pau), Spain

Thanks for your comment, and for the opportunity to clarify this issue. A thorough exploration of heterogeneity, its causes and its impact on a metanalysis is always needed, regardless of whether the metanalysis is finally conducted. Section 10.10.3 in the Cochrane handbook (in line with reference 57 in the manuscript) lists a number of strategies to deal with heterogeneity, including the option of not doing a metanalysis if there is “considerable variation in results, and particularly if there is inconsistency in the direction of effect”.  Establishing a threshold of inconsistency based on I ^2^ (as done in the review by Ellis and colleagues) is one possible approach to assess when the variation in results is too large to obtain a meaningful metanalysis estimation. While we have chosen to keep this example in the manuscript, we’ve modified the text to clarify that their use of I ^2^ was part of a more general assessment of heterogeneity and avoid the emphasis on thresholds.

# Peer review: Reviewer response for version 2

**DOI:** 10.5256/f1000research.28216.r69216

**Published:** 2020-09-01

**Authors:** Miranda Cumpston

**Affiliations:** 1 Monash University, Melbourne, Australia; 2 University of Newcastle, Newcastle, Australia

Thanks to the authors for their work in revising this paper and responding effectively to the comments raised in peer review. I have no further comments to raise.

 Thanks also for including me in the Acknowledgements - please note that my correct title is 'Ms'.

Is the work clearly and accurately presented and does it cite the current literature?

Yes

If applicable, is the statistical analysis and its interpretation appropriate?

Not applicable

Are all the source data underlying the results available to ensure full reproducibility?

No

Is the study design appropriate and is the work technically sound?

Yes

Are the conclusions drawn adequately supported by the results?

Yes

Are sufficient details of methods and analysis provided to allow replication by others?

No

Reviewer Expertise:

In presenting my comments, I acknowledge that my own experience focuses on methods around systematic reviews of the effects of interventions, and so I cannot comment with expertise on the details of methods described for other review types.

I confirm that I have read this submission and believe that I have an appropriate level of expertise to confirm that it is of an acceptable scientific standard.

# Peer review: Reviewer response for version 1

**DOI:** 10.5256/f1000research.24297.r59540

**Published:** 2020-02-28

**Authors:** Edward Purssell

**Affiliations:** 1 School of Health Sciences, City, University of London, London, UK

I have a few suggestions which may be helpful to the authors:

1. When discussing research methods, it may be worth just mentioning the efficacy/effectiveness issue.
2. I think that this "To obtain an efficient search with adequate sensitivity, performing searches in MEDLINE and EMBASE is sufficient, as they are the two most frequently used bibliographic databases ^39^, and they are enough to identify most relevant studies for a specific SR ^40^" might be a bit of a sweeping statement - are the two databases always sufficient? I think it would be helpful to mention some others as well - unless the authors really do believe that these are enough.
3. "involving a medical librarian can be convenient to improve the search quality ^47– 49^".  I am not sure that convenient is quite the right word.
4. Be clear to differentiate risk of bias at the study level from RoB at the review level. For example RoB2 is study-level, ROBIS is review level.
5. "For instance, in the SR by Ellis *et al.* (2017), the authors established a 70% heterogeneity limit for I ^2^, beyond which a meta-analysis combining the results would not be performed ^13^" - why 70%? This does not sound like a sensible decision making process anyway. Either the authors thought it was worth doing a MA in which case the heterogeneity form parts of the results, or it is not worth doing in which case this would not be a consideration. Also not doing a MA may have the effect of hiding this heterogeneity. There are also ways of investigating heterogeneity that can be illuminating.
6. Mention ROBIS and AMSAR2 as review-level tools.

Is the work clearly and accurately presented and does it cite the current literature?

Yes

If applicable, is the statistical analysis and its interpretation appropriate?

Not applicable

Are all the source data underlying the results available to ensure full reproducibility?

No

Is the study design appropriate and is the work technically sound?

Yes

Are the conclusions drawn adequately supported by the results?

Yes

Are sufficient details of methods and analysis provided to allow replication by others?

Partly

Reviewer Expertise:

Paediatrics, infection control, systematic review.

I confirm that I have read this submission and believe that I have an appropriate level of expertise to confirm that it is of an acceptable scientific standard, however I have significant reservations, as outlined above.

# Response

**Published:** 2020-07-19

**Authors:** Marta Roqué

**Affiliations:** 1- Iberoamerican Cochrane Centre - Sant Pau Biomedical Research Institute (IIB-Sant Pau), Spain

- When discussing research methods, it may be worth just mentioning the efficacy/effectiveness issue.

ANSWER: We agree that this is an important issue, which merits a full discussion that unfortunately falls outside the scope of this project. The selection of resources done is not dependent on whether the reviewer explores efficacy or effectiveness, and will be useful to the researchers regardless of their intended purpose. However, we have clarified this issue throughout the text by referring to 'effects of interventions' (rather than efficacy of interventions) and we have explicitly commented on it in the discussion.We have substituted 'efficacy of interventions' by 'effects of interventions'. We have added the following limitation "The selection of resources done is not dependent on whether the reviewer explores questions on efficacy or effectiveness, which are often described as explanatory or pragmatic questions, and will be useful to the researchers regardless of their intended purpose. However, we have not considered the resources to conduct in-depth exploration of effectiveness issues such as reviews of complex interventions or implementation reviews. "

- I think that this "To obtain an efficient search with adequate sensitivity, performing searches in MEDLINE and EMBASE is sufficient, as they are the two most frequently used bibliographic databases39, and they are enough to identify most relevant studies for a specific SR40" might be a bit of a sweeping statement - are the two databases always sufficient? I think it would be helpful to mention some others as well - unless the authors really do believe that these are enough.

ANSWER: Certainly, while MEDLINE and EMBASE are most used databases, there are other databases which can provide complementary information. However, the bibliographic references provided in the text supports the notion that these two databases are enough for an efficient search regardless of topic, and the role of these other databases is mostly complementary, but with little added benefit. To avoid the implicit suggestion that specialized databases cannot generate any added value, a sentence has been added mentioning them. Two sentences have been added "To obtain an efficient search with adequate sensitivity, performing searches in MEDLINE and EMBASE is may be sufficient especially in intervention reviews".  " These searches can be complemented with additional searches in other databases such as PEDro (Hyperlink: https://www.pedro.org.au/), which may provide specific information for certain topics. "

-  "involving a medical librarian can be convenient to improve the search quality47–49".  I am not sure that convenient is quite the right word.

ANSWER: Thanks, we've substituted the term 'convenient' for 'desirable' 

- Be clear to differentiate risk of bias at the study level from RoB at the review level. For example RoB2 is study-level, ROBIS is review level.

ANSWER: Thanks for pointing out this issue, we have clarified in the risk of bias section that this project focuses in presenting study-level resources, and that tools for reviews are not included in the manuscript. As we expand later in comment 6, we have been more explicit about the exclusion of overviews in the eligibility criteria section, and we have added a mention to this fact in the discussion section, as a limitation of the review, with an explicit mention of these tools. A sentence has been added "Assessing the risk of bias of the included studies is a key element in any SR". We have modified the eligibility criteria ("We excluded the methodological resources to develop other types of SRs (methodological, economic evaluation and qualitative research SRs, or overviews)" and the discussion ("Reviews of reviews (or overviews) were also not considered, and as such, there are a number of review-level resources which have not been discussed, for example the risk of bias assessment tool ROBIS or the methodological tool AMSTAR2")

- "For instance, in the SR by Ellis et al. (2017), the authors established a 70% heterogeneity limit for I2, beyond which a meta-analysis combining the results would not be performed13" - why 70%? This does not sound like a sensible decision making process anyway. Either the authors thought it was worth doing a MA in which case the heterogeneity form parts of the results, or it is not worth doing in which case this would not be a consideration. Also not doing a MA may have the effect of hiding this heterogeneity. There are also ways of investigating heterogeneity that can be illuminating.

ANSWER: We agree with the peer reviewer that the analysis choices of the examples shown may not always be universally shared. We chose the examples based on several considerations, as they had to be useful for the different parts of the manuscript, but they are not necessarily a perfect role model in all their decisions. Readers will need to take into account that these examples are only illustrative, as we are presenting these choices descontextualized, without a full vision of the authors decision making process. 

- Mention ROBIS and AMSTAR2 as review-level tools.

ANSWER:  Thanks for this suggestion. We restricted our focus to a limited list of SR types, not including overviews (or reviews of reviews). For this reason, only study-level resources for assessing risk of bias have been described, and review-level resources (such as ROBIS or AMSTAR2) are not mentioned. We realize that this issue may not be clear enough, and consequently we have been more explicit about the exclusion of overviews in the eligibility criteria section, and we have added a mention to this fact in the discussion section, as a limitation of the review, with an explicit mention of these tools. We have modified the eligibility criteria ("We excluded the methodological resources to develop other types of SRs (methodological, economic evaluation and qualitative research SRs, or overviews)" and the discussion ("Reviews of reviews (or overviews) were also not considered, and as such, there are a number of review-level resources which have not been discussed, for example the risk of bias assessment tool ROBIS or the methodological tool AMSTAR2")

# Peer review: Reviewer response for version 1

**DOI:** 10.5256/f1000research.24297.r59538

**Published:** 2020-02-14

**Authors:** Miranda Cumpston

**Affiliations:** 1 Monash University, Melbourne, Australia; 2 University of Newcastle, Newcastle, Australia

The authors have summarised the current literature around methods for conducting systematic reviews, encompassing reviews of prevalence, prognosis, diagnosis and intervention effectiveness. In a field of methodology where some guidance is collated in well-known handbooks, but other areas are scattered among the methods literature, and where development of new methods is relatively fast-moving, this map of available guidance and methods studies is likely to be very useful to researchers new to systematic reviews or branching out into different review types. The authors are experienced experts in this field, and the review is well-written and clear.

 In my understanding, this paper aims to fulfil two roles: (1) a toolkit that enables authors of systematic reviews to navigate out to key methods resources, and (2) a brief summary of methods guidance for each review type. It may be helpful throughout the paper, including in the abstract and conclusions, to separate out these two roles more clearly. I have some suggestions to improve the transparency and usability of the paper from each of these perspectives.

**Major recommendations**

 As a review and toolkit of methodological resources, I would make the following suggestions:

- **Methods: **The authors could provide more transparent detail on the selection process for included documents, including specifying which organisational websites were searched, how many resources were identified, and if any were excluded. Although this is intended as a mapping, rather than systematic review, some additional detail would help readers to understand how the guidance documents were selected, and why perhaps some resources they may have used before are not listed.
- Importantly, more detail could presented on how the authors selected the most “relevant” or “best” resources, especially where there may have been multiple candidate documents. For example, were they most recent, most comprehensive, those endorsed by a credible organisation, most introductory, most rigorous methods? The role of the authors’ expert judgement in these decisions should be stated explicitly. Note that I am not suggesting that a different process should have been used, just suggesting a more detailed description of the judgement process.
- **Results: **As a navigation guide, I found that citing the resources within the text did not tell me everything I wanted to know. Also, the key resources are mixed in the reference list with exemplar reviews and other citations. A table outlining the recommended resources by category with hyperlinks to each resource would be helpful.
- As a reader, I would find it helpful for the authors to draw the distinction between the different types of resources cited. For example, some were synthesised, ‘best practice’ guidance intended for use by authors (such as the Cochrane or JBI Handbooks). Some were reporting guidelines (which often list good practice but are not intended to provide detailed guidance on the conduct of reviews). Some were primary methods studies (e.g. measuring the prevalence or impact of a method, which may describe or evaluate possible methods options but are not intended as guidance on the ‘best’ available methods for authors. All may be useful in different ways.

As a brief summary of methods guidance, I would make the following suggestions:

- **Methods: **It would be helpful to include a brief description of your role in selecting and writing this summary of guidance, for example, stating briefly that key areas of methods were summarised, and how the methods to be highlighted were selected, especially where multiple and potentially conflicting sources were available (e.g. major methods applicable to all reviews, key differences between review types, expert opinion about interesting or important advice).
- It might be helpful to note that the summary of guidance presented is just that, a brief summary, and that authors who are new to systematic reviews should consult the more detailed documents for complete guidance.
- **Results: **It might be particularly helpful to note where there is disagreement in the literature in relation to particular areas of guidance. For example, comments that searching the grey literature is not useful for effectiveness reviews, or that risk of bias tools should be adapted for each review, which may be in disagreement with some of the major guidance handbooks and reporting guidelines. I’m not arguing about these specific items, just noting that some methods choices have been made by the authors, and it may be helpful to make this transparent.

**Minor recommendations**

- **Title and abstract: **It would be helpful to briefly define the scope of this review, especially in the context of a journal such as F1000Research, which publishes in a wide range of scientific fields. For example, using a term such as ‘systematic reviews in health care’, and noting that the review looks at prevalence, prognosis, diagnosis and health interventions (and not other areas relevant to health such as environmental exposure).
- **Competing interests: **The authors could note any roles with Cochrane and the GRADE Working Group. These are not necessarily financial conflicts, but may be relevant to the authors’ decision to recommend guidance from these sources (which of course I agree with!).
- **Results: **In the first two paragraphs, you discuss some specific points in relation to resources available for diagnostic and prognosis reviews. As all review types appeared to cite both guidance handbooks and additional primary methods studies, I wasn’t clear on the point you were trying to make in this section.
- There were some key guidance documents that I would have expected to see, although there may be good reasons not to include them, such as:
  - General guidance on systematic reviews from the US Institute of Medicine and the Centre for Reviews and Dissemination at the University of York (both are older documents, so this may be the reason).
  - Tools to assess the risk of bias in reviews, such as ROBIS or AMSTAR, which may enable reflection by authors on their choice of methods, in a similar manner to reporting guidelines.
  - Resources on the development of protocols (as distinct from study registration), such as Chapter 1 of the Cochrane Handbook.
  - Guidance on synthesis in the absence of meta-analysis, such as Chapter 12 in the Cochrane Handbook and papers by (Campbell, McKenzie *et al*. (2020) ^1^) and (Popay, Roberts *et al*. (2006) ^2^). This may be relevant to synthesis, assessment of heterogeneity and GRADE.
  - Guidance on network meta-analysis, such as Chapter 11 of the Cochrane Handbook and multiple journal articles, as well GRADE guidance and CINeMA.
  - Two papers from the GRADE Working Group (published this week!) on the use of GRADE for diagnostic test accuracy studies published this week.
- In the text on risk of bias and Table 2, is it worth noting that the number of items in RoB 2 varies depending on the effect of interest and the included study designs?
- In Table 3, as there are more variations on the measures to combine (e.g. Ratio of Means), and statistical methods for meta-analysis of efficacy of interventions (including the inverse-variance method, which is mentioned for other types), could it be helpful to note in the table somewhere that these are common characteristics, not an exhaustive list?
- When discussing fixed effect and random effects models, it may be helpful to note that they differ in relation to assumptions and heterogeneity, as you mention something briefly about analysis using random-effects models later in the paper that relies on understanding this.
- On page 9, under “Efficacy of intervention reviews”, I think hazard ratios are listed in error against binary outcomes as well as time-to-event outcomes. Perhaps you meant risk ratios?
- In the section on assessing certainty of the evidence, it would be helpful to describe the GRADE approach in the first paragraph (it is currently named without description in the third paragraph).
- **Discussion:** I would also acknowledge under limitations of this review that the selection of resources and summary of guidance were informed by expert opinion, and that others may have selected different resources or made different recommendations.
- **References: **1: I think there may be an error – should this be a reference to the 2019 edition of the Cochrane Handbook, rather than an older paper by Higgins in *Cochrane Methods* on RoB 2? I assume this based on its use in the Introduction as a general reference about review methods, and also on the availability of general Handbooks in para 2 of the Results.
- 20: could be based on the references to individual chapters, i.e. Deeks JJ, Bossuyt PM, Gatsonis C (editors), Cochrane Handbook for Systematic Reviews of Diagnostic Test Accuracy.
- 63: It’s great to see the Spanish Manual GRADE cited. If I am correct, this is a translation of the 2013 GRADE Handbook (apologies if I am wrong). Would it be helpful to cite some of the more recent GRADE papers as well, as they reflect the most up to date guidance? I note you also cite the paper on using standard language to express GRADE, as well as the recent Cochrane Handbook Chapter, so perhaps this is sufficient.

Is the work clearly and accurately presented and does it cite the current literature?

Yes

If applicable, is the statistical analysis and its interpretation appropriate?

Not applicable

Are all the source data underlying the results available to ensure full reproducibility?

No

Is the study design appropriate and is the work technically sound?

Yes

Are the conclusions drawn adequately supported by the results?

Yes

Are sufficient details of methods and analysis provided to allow replication by others?

No

Reviewer Expertise:

In presenting my comments, I acknowledge that my own experience focuses on methods around systematic reviews of the effects of interventions, and so I cannot comment with expertise on the details of methods described for other review types.

I confirm that I have read this submission and believe that I have an appropriate level of expertise to confirm that it is of an acceptable scientific standard, however I have significant reservations, as outlined above.

## References

[1] : Synthesis without meta-analysis (SWiM) in systematic reviews: reporting guideline. *BMJ*.2020; 10.1136/bmj.l6890 10.1136/bmj.l6890 PMC719026631948937

[2] : Guidance on the conduct of narrative synthesis in systematic reviews. *ESRC methods programme*.2006;47-71

# Response

**Published:** 2020-07-19

**Authors:** Marta Roqué

**Affiliations:** 1- Iberoamerican Cochrane Centre - Sant Pau Biomedical Research Institute (IIB-Sant Pau), Spain

-  Methods: The authors could provide more transparent detail on the selection process for included documents, including specifying which organisational websites were searched, how many resources were identified, and if any were excluded.

ANSWER: Thanks for this suggestion. The text lists the main organisational websited we checked to identify guidelines ("Cochrane, Joanna Briggs Institute, European Network for Health Technology Assessment (EUNETHTA), Enhancing the Quality and Transparency of Health Research (EQUATOR) network, Grading of Recommendations Assessment, Development and Evaluation (GRADE))", as suggested by the peer reviewer. Also, the text is now more explicit about the process followed to select resources. See answers to comments below regarding this specific issue.More detailed description of the resource selection process has been added to the text (see detailed modifications in the next comments)

-  Importantly, more detail could presented on how the authors selected the most “relevant” or “best” resources, especially where there may have been multiple candidate documents.

ANSWER: Thanks for this suggestion. A paragraph was added to the eligibility criteria section "The resources were selected based on the authors expert judgement, prioritising those resources which were endorsed or part of a guideline from the organisations cited above, and those which were more recent. "

- Results: As a navigation guide, I found that citing the resources within the text did not tell me everything I wanted to know.

ANSWER: We have added a table in the Appendix where the references and resources considered in the review are classified into guidelines to conduct SR (or chapters of those guidelines), reporting guidelines, primary methods papers and references to examples used in the manuscript.

-  Methods: It would be helpful to include a brief description of your role in selecting and writing this summary of guidance, 

ANSWER: Thanks, we will be more explicit on this issue. A sentence has been added to the data selection and extraction section "For each pre-defined section, the authors selected and summarized the methods that were considered to be more rigorous and widely accepted, prioritizing major methods applicable to all reviews over more controversial methods, or methods which required highly specialized knowledge. The text organises the results pedagogically with the aim to highlight key differences between review types, present the key characteristics of each method, and be a comprehensive tool that contains the most relevant advice based on the authors judgement."

-  It might be helpful to note that the summary of guidance presented is just that, a brief summary, and that authors who are new to systematic reviews should consult the more detailed documents for complete guidance.

ANSWER: Thanks for the suggestion, we have clarified this point in the text. A sentence has been added to the key results section of the discussion, stating "This project does not aim to be a standalone tool for a researcher to find complete guidance on how to conduct and report a review, but rather it aims to be a signpost pointing out to the resources where researchers may find in depth guidance to develop their reviews."

- Results: It might be particularly helpful to note where there is disagreement in the literature in relation to particular areas of guidance.

ANSWER: As far as possible, we have avoided presenting controversial advice, although we are aware that any topic can be approached differently by different researchers or even institutions. It could be quite daunting to be comprehensive in identifying all the controversies in the different steps of conducting a review, as the volume of publications on methods for reviews is extremely large and diverse. Additionally, we think that the discussion or even the identification of issues where controversy exists may fall outside of the project scope, as it might reduce the usefulness to a new researcher which needs to find clear  guidance on a topic, even if there are other alternative methods available.  We have stressed the subjectiveness and risk of implicit selection biases in the discussion "An inherent limitation of this project is its methodology based on a selection of resources and summary of guidance informed by expert opinion, which may be susceptible to implicit selection biases or lack of comprehensiveness. "

- Title and abstract: It would be helpful to briefly define the scope of this review, especially in the context of a journal such as F1000Research, which publishes in a wide range of scientific fields.

ANSWER: Thanks, this is a very useful suggestion.Title has been modified to "Toolkit of methodological resources to conduct systematic reviews in health care: reviews on prevalence, prognosis, diagnosis and interventions". The concept has also been introduced in the abstract ("This work identifies and describes the most relevant methodological resources to conduct high-quality reviews that answer health care questions regarding prevalence, prognosis, diagnostic accuracy and effects of interventions")

-  Competing interests: The authors could note any roles with Cochrane and the GRADE Working Group.

ANSWER: A sentence was added to the Data selection and extraction "The authors are members of CIBERESP (Centro de Investigación Biomédica en Red de Epidemiología y Salud Pública - Biomedical Research Center Network of Epidemiology and Public Health), hold active roles within Cochrane and the GRADE Working Group,"

-  Results: In the first two paragraphs, you discuss some specific points in relation to resources available for diagnostic and prognosis reviews. As all review types appeared to cite both guidance handbooks and additional primary methods studies, I wasn’t clear on the point you were trying to make in this section.

ANSWER: We realize we didn't make our point clear, that was to illustrate what guideline handbooks had been identified by type of review. We've rewritten both paragraphs aiming to be more clear. "We identified guidance handbooks, primary studies and reporting guidelines as a result of the bibliographic searches. The resources selected are presented in the Appendix.

We have identified methodological guidelines dedicated to the development of prevalence SRs14, global prognosis15, and prognostic factor SRs 16, 17, 18 .

During the performed search, we identified methodological manuals to develop prognostic model SRs in the series of publications from the PROGRESS project19, and in the resource compilation from Cochrane's Prognosis Methods Group  ."

- There were some key guidance documents that I would have expected to see,

ANSWER: .Thanks for the bringing forth this issue, which was overlooked in the manuscript. The issue of narrative synthesis of results has been cited in several sections the paper (statistical synthesis, quality of evidence, results report). An explicit mention to narrative synthesis has been added to the Statistical synthesis section "In those cases when a quantitative synthesis is precluded, the SR will be restricted to a narrative synthesis. A narrative synthesis should not simply summarize the findings from the included studies in order to draw conclusions about the body of evidence, but instead should be a more formal process which includes a formulation of the theory of how the intervention works, why and for whom, the exploration of the relationships in the data, and the assessment of the robustness of the synthesis. 59". Certainty of evidence from narrative synthesis has been mentioned "Certainty of evidence can be assessed too when no quantitative synthesis is possible. 65".  Also, the SWiM paper has been cited in the REsults report section"Additionally,  the SWiM guideline is available for reporting intervention SRs where the effects of interventions are synthethised narratively without metanalysis, focusing on the key features of narrative information synthesis (grouping of studies, presentation of data and summary text, and appropriate discussion of limitations of this type of synthesis)."

Network metanalysis have been mentioned in the Statistical methods and Quality of evidence sections. A cite to Chapter 11 in the handbook has been added to both sections

Thanks, we've added the GRADE diagnostic  references to the paper. These references were incorporated to the corresponding Quality of evidence section, modifying the paragraphs on diagnostic SR.

- In the text on risk of bias and Table 2, is it worth noting that the number of items in RoB 2 varies depending on the effect of interest and the included study designs?

ANSWER: Thanks, we've added this information. A sentence has been added to the risk of bias section ("The number of questions may vary, depending on the effect of interest and the design of the study assessed"). A footnote has been added to Table 2 "The number of items in the risk of bias tools may vary depending on the effect of interest and the included study designs, as well as the addition or suppresion of index questions by the researchers to tailor the tool to the SR ."

-  In Table 3, as there are more variations on the measures to combine (e.g. Ratio of Means), and statistical methods for meta-analysis of efficacy of interventions (including the inverse-variance method, which is mentioned for other types), could it be helpful to note in the table somewhere that these are common characteristics, not an exhaustive list?

ANSWER: Thanks, we've corrected the oversight of not mentioning the inverse variance method for continuous outcomes in the efficacy of interventions SR, and have clarified in the text that the table is by no means exhaustive. The text introducing table 3 now reads "Table 3 shows a non-exhaustive compilation of the main characteristics of the meta-analysis methods and the main software commands for each type of SR". The inverse-variance method has been added to the efficacy of interventions row

- When discussing fixed effect and random effects models, it may be helpful to note that they differ in relation to assumptions and heterogeneity, as you mention something briefly about analysis using random-effects models later in the paper that relies on understanding this.

ANSWER: This is certainly an important point. Although there is already an explicit link between choice of model and heterogeneity in the previous paragraph ("the evaluation of the existing clinical and statistical heterogeneity in the set of studies, which will inform us /.../ 2) what meta-analysis model we should apply"), we agree to further stress this point as suggested. We've modified the existing sentence on random and fixed-effects models "Both models are based on different assumptions regarding distribution of effects and heterogeneity across studies, and they differ in their application and interpretation" 

- On page 9, under “Efficacy of intervention reviews”, I think hazard ratios are listed in error against binary outcomes as well as time-to-event outcomes. Perhaps you meant risk ratios?

ANSWER: You're right, it should have read 'risk ratios' instead of 'hazard ratios'. hazard ratio' substituted by 'risk ratio'

-  In the section on assessing certainty of the evidence, it would be helpful to describe the GRADE approach in the first paragraph (it is currently named without description in the third paragraph).

ANSWER: Thanks, we agree. We've added an explicit reference to the GRADE system "Certainty of evidence is best evaluated with the GRADE system . Certainty in the obtained estimates for each one of the key SR outcomes or factors is classified as high, moderate, low or very low"

- Discussion: I would also acknowledge under limitations of this review that the selection of resources and summary of guidance were informed by expert opinion, and that others may have selected different resources or made different recommendations.

ANSWER: While we have strived to draw a unbiased selection of the best resources, it is right to point out this potential limitation. We've added the sentence "An inherent limitation of this project is its methodology based on a selection of resources and summary of guidance informed by expert opinion, which may be susceptible to implicit biases or lack of comprehensiveness"

-  Reference 1: I think there may be an error – should this be a reference to the 2019 edition of the Cochrane Handbook, rather than an older paper by Higgins in Cochrane Methods on RoB 2?

ANSWER: Yes, you are right. References 1 and 56 were interchanged, and have now been corrected. New reference 1 and new reference 56

- Reference 20: could be based on the references to individual chapters, i.e. Deeks JJ, Bossuyt PM, Gatsonis C (editors), Cochrane Handbook for Systematic Reviews of Diagnostic Test Accuracy.

ANSWER: Thank you, the reference has been modified as suggested

- Reference 63: It’s great to see the Spanish Manual GRADE cited. If I am correct, this is a translation of the 2013 GRADE Handbook (apologies if I am wrong). Would it be helpful to cite some of the more recent GRADE papers as well, as they reflect the most up to date guidance? I note you also cite the paper on using standard language to express GRADE, as well as the recent Cochrane Handbook Chapter, so perhaps this is sufficient.

ANSWER: We wish to keep the original 2013 reference, as the most comprehensive manual on GRADE. However, we will substitute the reference of the Spanish version for the English version. Reference 63 substituted for the reference to the original version
